# Supplementary material for: Contribution of retrotransposition to developmental disorders
Source: Nat Commun. 2019 Oct 11;10:4630. doi: 10.1038/s41467-019-12520-y (PMC6789007; doi:10.1038/s41467-019-12520-y)
Supplement: Supplementary file 1 — Supplementary Information [file 41467_2019_12520_MOESM1_ESM.pdf]

Supplementary Information for “Contribution of Retrotransposition to Developmental Disorders”

Eugene J. Gardner[1], Elena Prigmore[1], Giuseppe Gallone[1], Petr Danecek[1], Kaitlin E. Samocha[1], Juliet Handsaker[1], Sebastian S. Gerety[1], Holly Ironfield[1], Patrick J. Short[1], Alejandro Sifrim[2], Tarjinder Singh[1], Kate E. Chandler[3], Emma Clement[4], Katherine L. Lachlan[5,6], Katrina Prescott[7], Elisabeth Rosser[4], David R. FitzPatrick[8], Helen V. Firth[1,9], and Matthew E. Hurles[1,a]

[1]Wellcome Trust Sanger Institute, Wellcome Genome Campus, Cambridge, Hinxton, CB10 1SA, United Kingdom
[2]Department of Human Genetics, Herestraat 49, Box 602, B-3000, Leuven, Belgium
[3]Manchester Centre for Genomic Medicine, Manchester University Hospitals NHS Foundation Trust, Manchester Academic Health Science Centre, Manchester, Greater Manchester, M13 9WL, United Kingdom
[4]Department of Clinical Genetics, North East Thames Regional Genetics Service, Great Ormond Street Hospital for Children NHS Trust, Holborn, London, WC1N 3JH, United Kingdom
[5]Wessex Clinical Genetics Service, Southampton University Hospitals NHS Foundation Trust, Princess Anne Hospital, Southampton, SO16 5YA, United Kingdom
[6]Faculty of Medicine, Human Development and Health, University of Southampton, Southampton, SO17 1BJ, United Kingdom
[7]Chapel Allerton Hospital, Yorkshire Regional Genetics Service, Leeds Teaching Hospitals NHS Trust, Leeds, LS7 4SA, United Kingdom
[8]MRC Human Genetics Unit, MRC IGMM, University of Edinburgh, WGH, Edinburgh, EH4 2SP, United Kingdom
[9]East Anglian Medical Genetics Service, Box 134, Cambridge University Hospitals NHS Foundation Trust, Cambridge Biomedical Campus, Cambridge CB2 0QQ, United Kingdom

[a] meh@sanger.ac.uk

Supplemental Figure 1

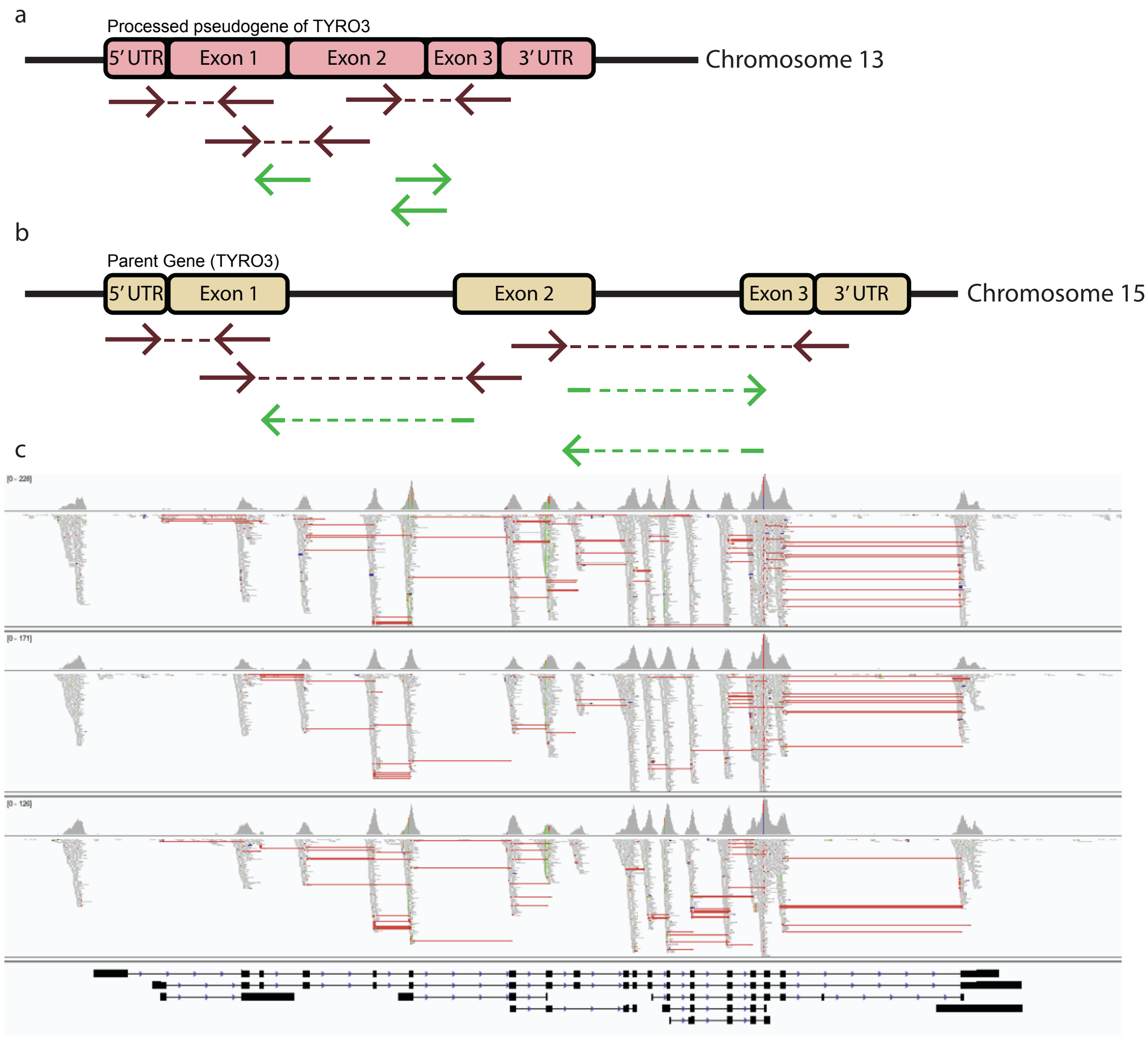

Identification of processed pseudogenes (PPGs). Included is an example of the read-pair evidence utilized by our PPG discovery pipeline. Shown throughout the figure is how read evidence supporting the duplication of *TYRO3* aligns to: (**a**) a cartoon example of the PPG, (**b**) a cartoon of the donor gene, and (**c**) a screenshot of read-pair evidence supporting the duplication aligned to *TYRO3* for one trio viewed in the Integrative Genomics Viewer (IGV)[1]. For the cartoons in (**a**) and (**b**), split read pairs (SRPs) are shown in green, with discordant read pairs (DRPs) shown in dark red. When aligned to the duplication, SRPs and DRPs align without gaps and/or clipped sequence. When aligned to the source gene as in (**b**), these reads align further apart than expected or have clipped ends (long dashed lines). This is shown in real data in (**c**), where the red lines represent DRPs that have a larger insert size than the WES library preparation.

Supplemental Figure 2

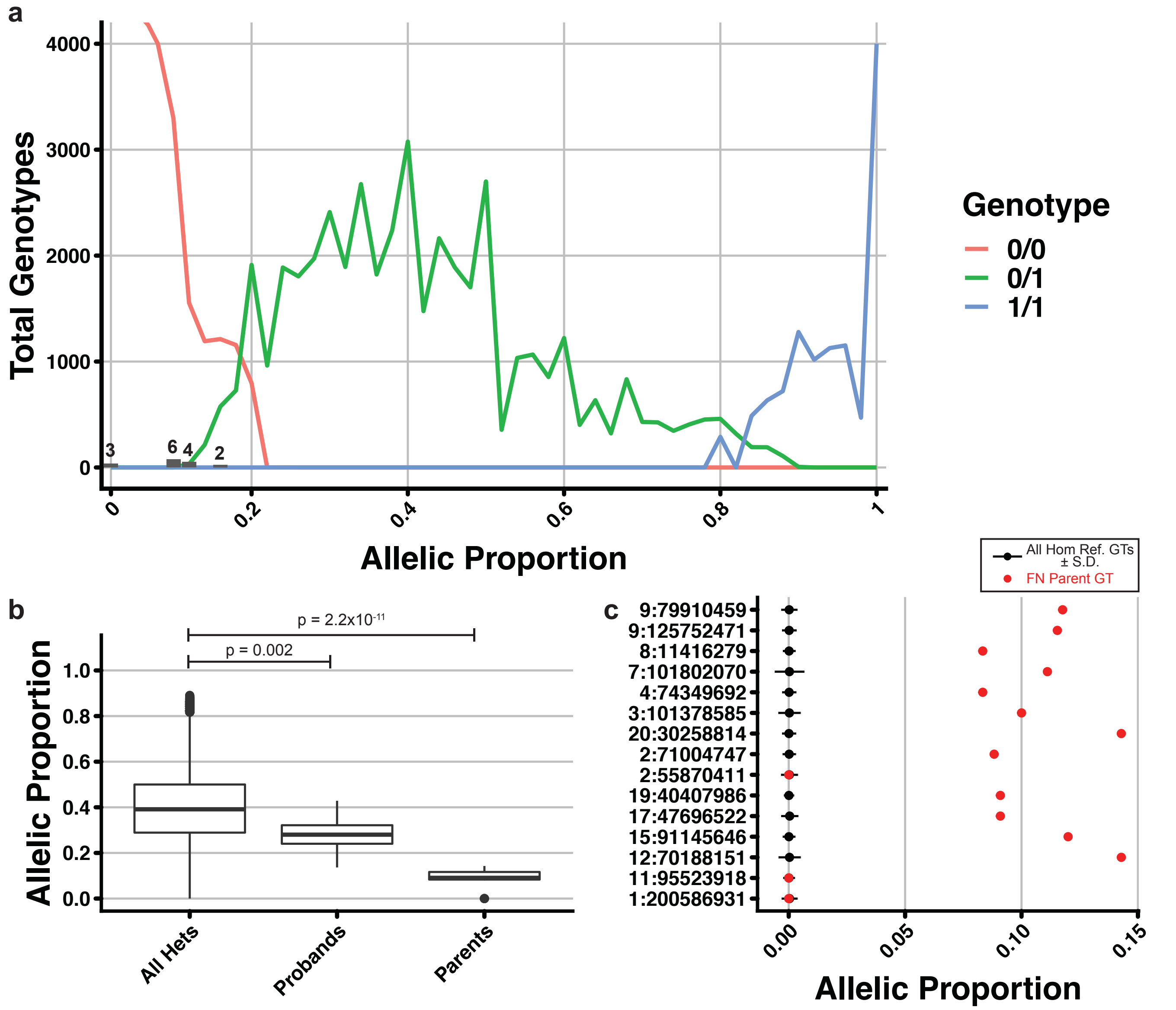

Allelic proportion of *Alu* variants in the DDD study. (**a**) Calculation of allelic proportion from a subset of DDD trios. Displayed are a total of 2,532,531 non-fail (./.) genotypes from the 917 *Alu* sites identified in this study with a depth of at least 10X coverage separated into 0.02 allelic proportion bins for 1,000 randomly selected and 15 false negative trios. Allelic proportion was calculated as number of insert supporting reads / total reads spanning the insertion site. Lines are coloured based on the MELT-assigned genotype (hom ref [*0/0*], het [*0/1*], hom alt [*1/1*] as red, green, blue, respectively). Note that, due to the excess of homozygous reference genotypes, the line indicating total homozygous reference sites continues above the y-axis. *Alu* sites with a false negative genotype in a parent (i.e. initially thought to be *de novo*) are shown as grey bars with total number of sites in each bin shown above. As is shown by this plot, false negative genotypes do fall on the extreme end of true heterozygous genotypes, but also fall within other sites which are deemed homozygous reference by MELT genotyping. (**b**) Comparison of allelic proportion. Shown are Tukey boxplots of aggregate allelic proportion for all heterozygous variants re-genotyped in (**a**; All Hets), 15 heterozygous variants called as *de novo* but on manual inspection were likely inherited (Probands), and corresponding parents to this subset of probands (Parents). P-values abocve boxplots were calculated via a Wilcoxon rank-sum test. While both results are statistically significant and our potentially mosaic loci differ in allelic proportion to others in the genome, the true difference from the median allelic proportion of all heterozygous variants for parental genotypes (difference = 0.30) is clearly larger than that for MELT-called heterozygous probands (difference = 0.11). (**c**) Assessment of genotyping error at false negative loci. Black points (lines ± SD) are mean allelic proportion for all homozygous reference genotypes at each of the 15 assessed false negative *de novo* loci. Red points indicate the allelic proportion in each of the 15 false negative parents at the locus indicated on the y-axis. In this case, 12/15 (80%) false negative parents showed a difference from the homozygous allelic proportion distribution (z-test $p < 0.05$). Additionally, the individuals with homozygous reference genotypes at each locus do not show higher error rates and are closely centered around the expected proportion of 0.

Supplemental Figure 3

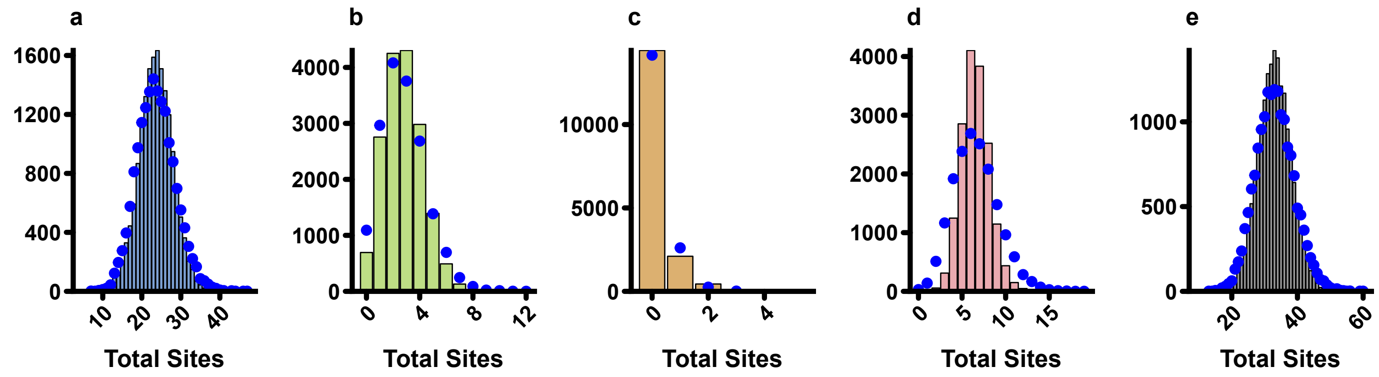

Poisson distribution projected onto allele counts per individual. Shown are identical plots to main text Fig. 1a-e for *Alu* (a), L1 (b), SVA (c), PPGs (d), and all RT events (e), but with Poisson distributions based on the mean number of sites per individual projected onto each (shown as blue points).

Supplemental Figure 4

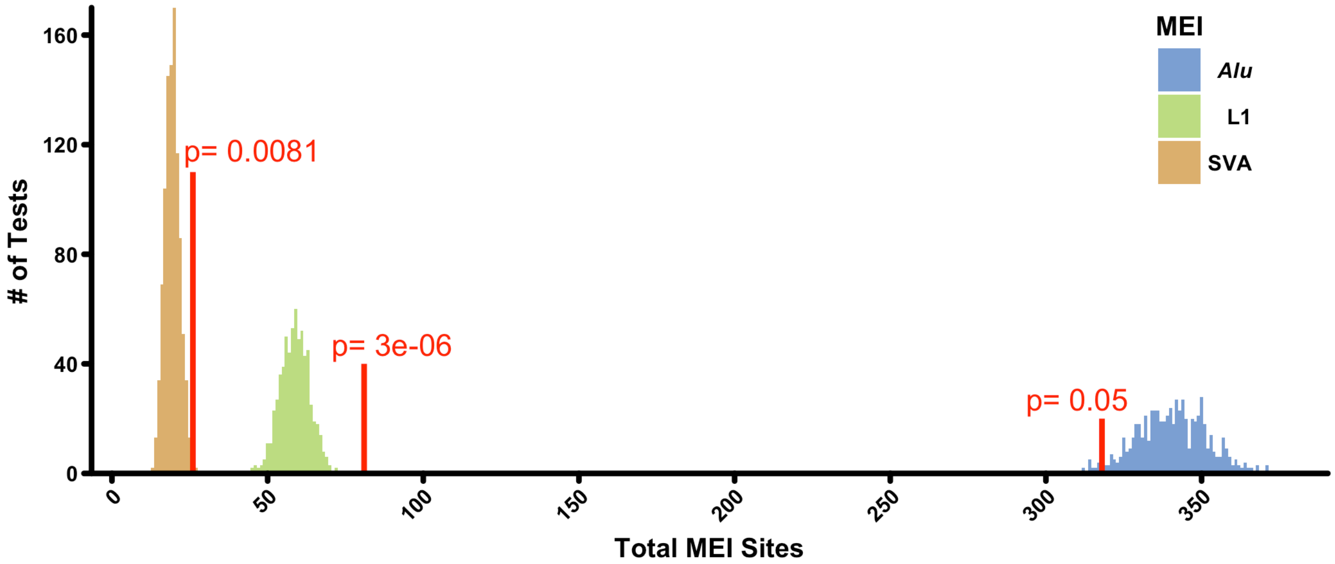

Downsample of DDD to 1KGP size. Shown are histograms for DDD data randomly down-sampled 1,000 times to the population size of the 1KGP cohort for all three MEI types (*Alu* – blue, L1 – green, and SVA – orange). Red lines indicate the total number of sites in the 1KGP within WES bait regions, with p-values derived from a z-score independently for each MEI class shown above.

Supplemental Figure 5

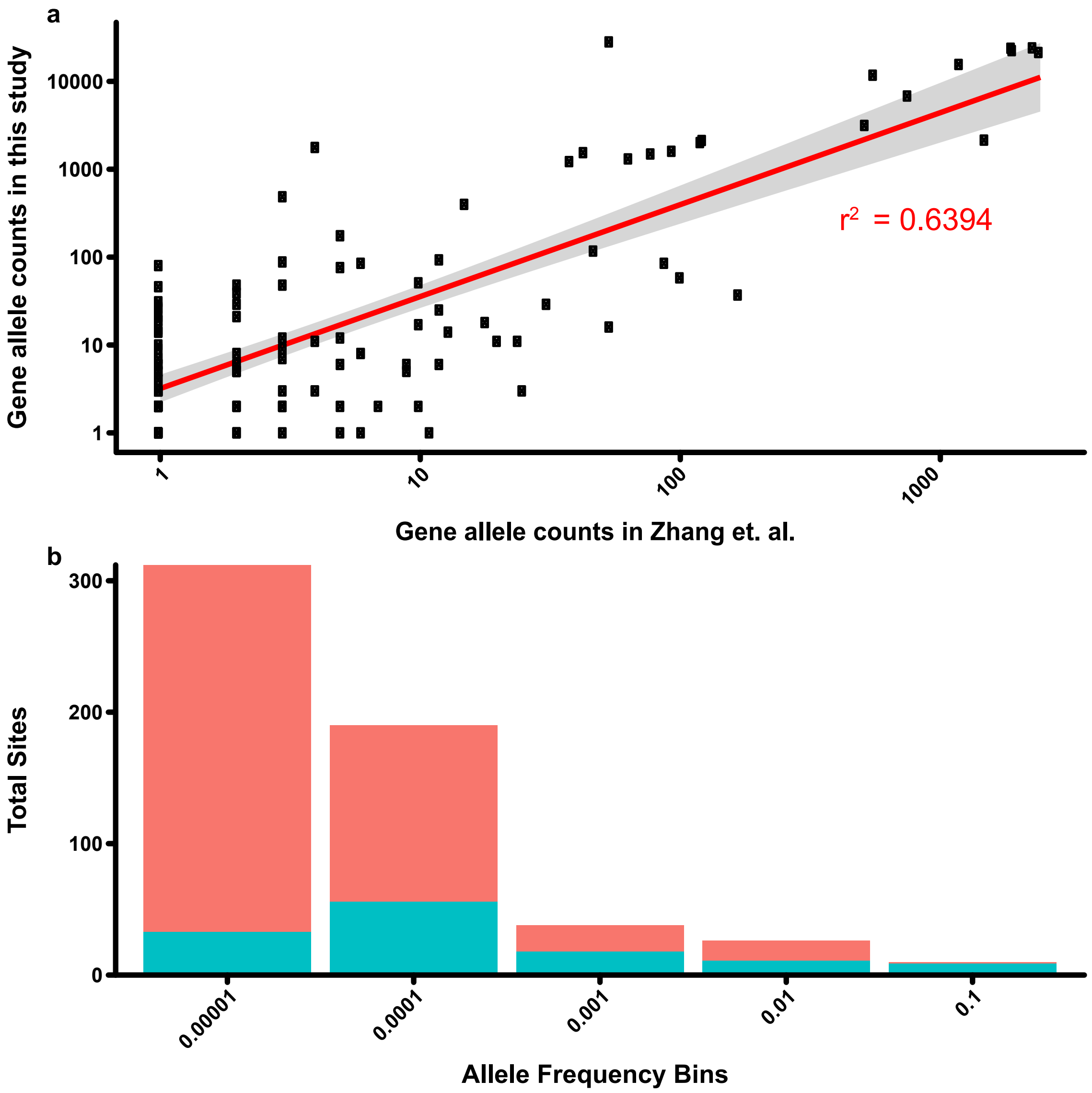

Comparison of Zhang et. al.[2] and this study. (**a**) Allele count comparison between Gardner & Zhang. Points represent total number of individuals in which a PPG was observed for Zhang et. al.[2] (x-axis) and this study (y-axis). Regression line is given in red, with calculated r^2 listed below. (**b**) Counts of known (light blue) and unknown (pink) PPGs separated into $\log_{10}$ allele frequency bins. The majority of rare PPGs (AF < 0.001) were identified only in this study.

Supplemental Figure 6

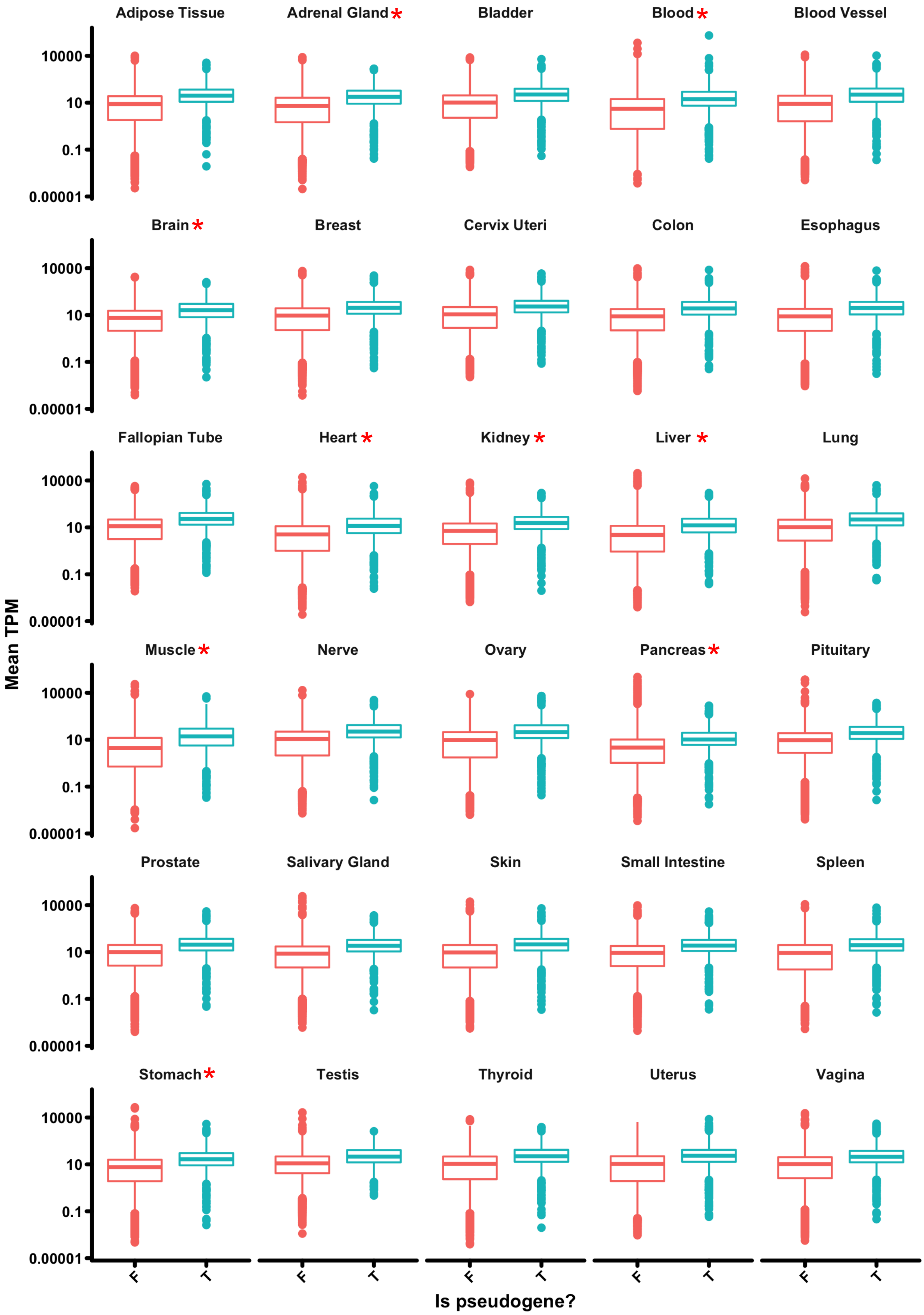

Donor gene expression in GTEx[3]. Shown for each tissue type are Tukey boxplots of the expression in TPM of donor genes that gave rise to duplications (T – light blue) and did not give rise to duplications (F – light red). Within each tissue, genes which gave rise to duplications have TPM values which are significantly higher (Wilcoxon rank-sum p-value < $1x10^{-3}$) than those of their non-duplicated counterparts. * - indicates tissues which have a Wilcoxon rank-sum p-value < $1x10^{-3}$ when compared to testis and ovary donor gene expression.

Supplemental Figure 7

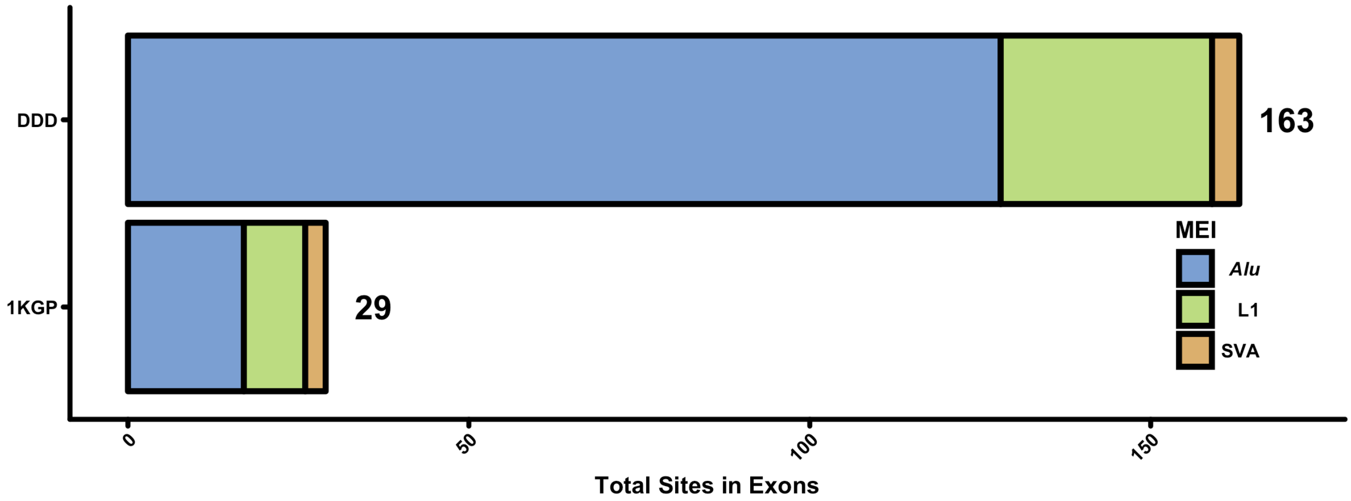

Total exonic variants identified in 1KGP and DDD. Shown are total exonic variants as identified in the 1KGP compared to those identified in all individuals included in this study (n = 28,132 individuals). Each bar plot is divided into the three ME classes analysed as part of this study (*Alu*, L1, and SVA).

Supplemental Figure 8

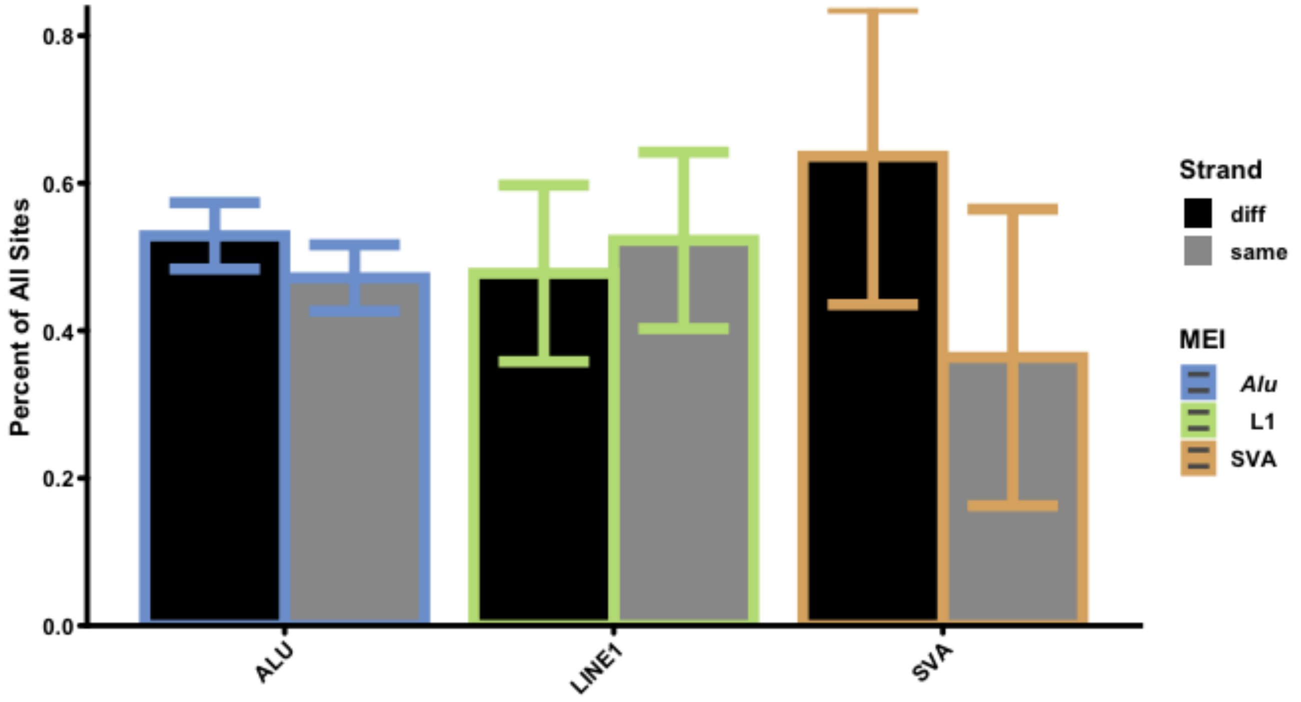

Strand bias data for intronic MEI sites. Percentage of all MEIs that are either in the same orientation (same – grey) or in opposite orientation (diff – black) as their host gene for all three ME types (*Alu* – blue, L1 – green, and SVA – orange). Error bars are 95% CI based on the population proportion. None of the differences shown are statistically significant at χ^2 p = 0.05.

Supplemental Figure 9

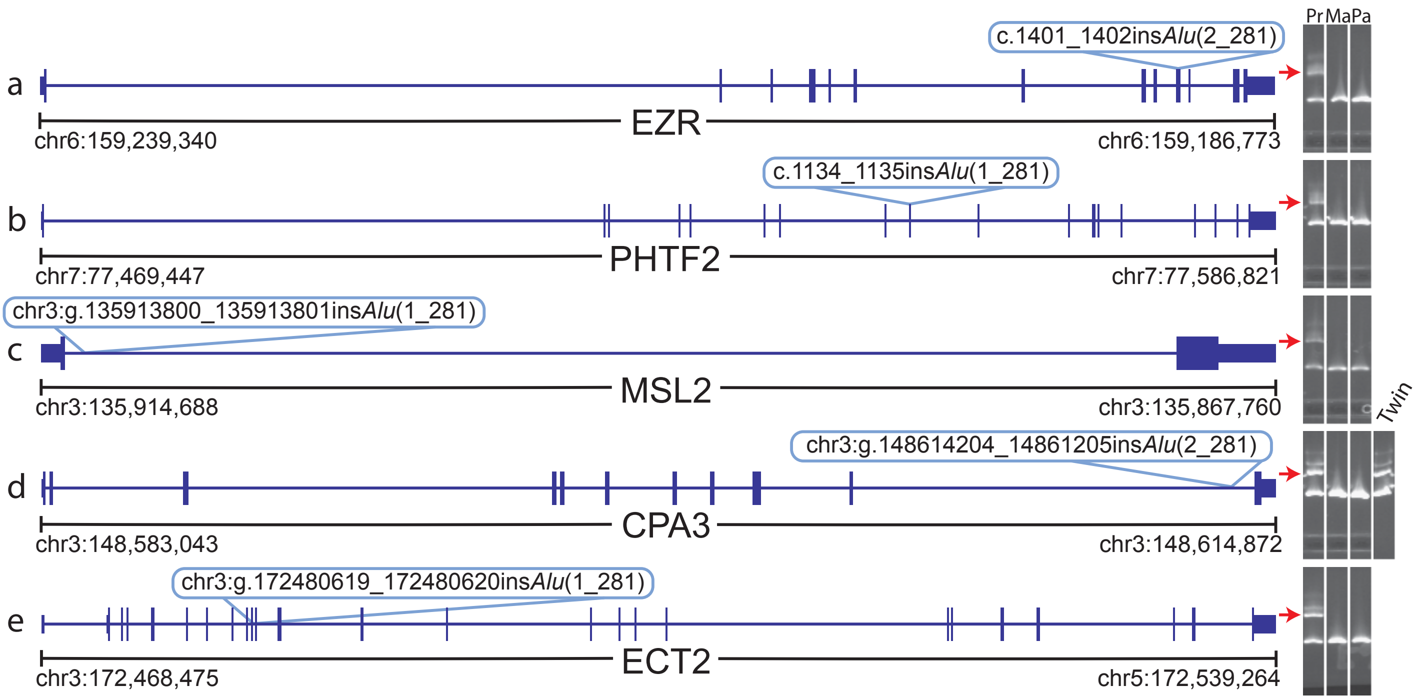

Non-clinically relevant de novo MEIs. Shown are plots identical to those in main text Fig. 3a-d, except for *de novo* MEIs that are not clinically actionable. Each gene is shown with site of impact with the colour of the bubble indicating the causal RT type. For CPA3 (**d**), additionally shown is the *de novo* MEI found in the proband's twin to the right of the proband (Pr), mother (Ma), and father (Pa).

Supplemental Figure 10

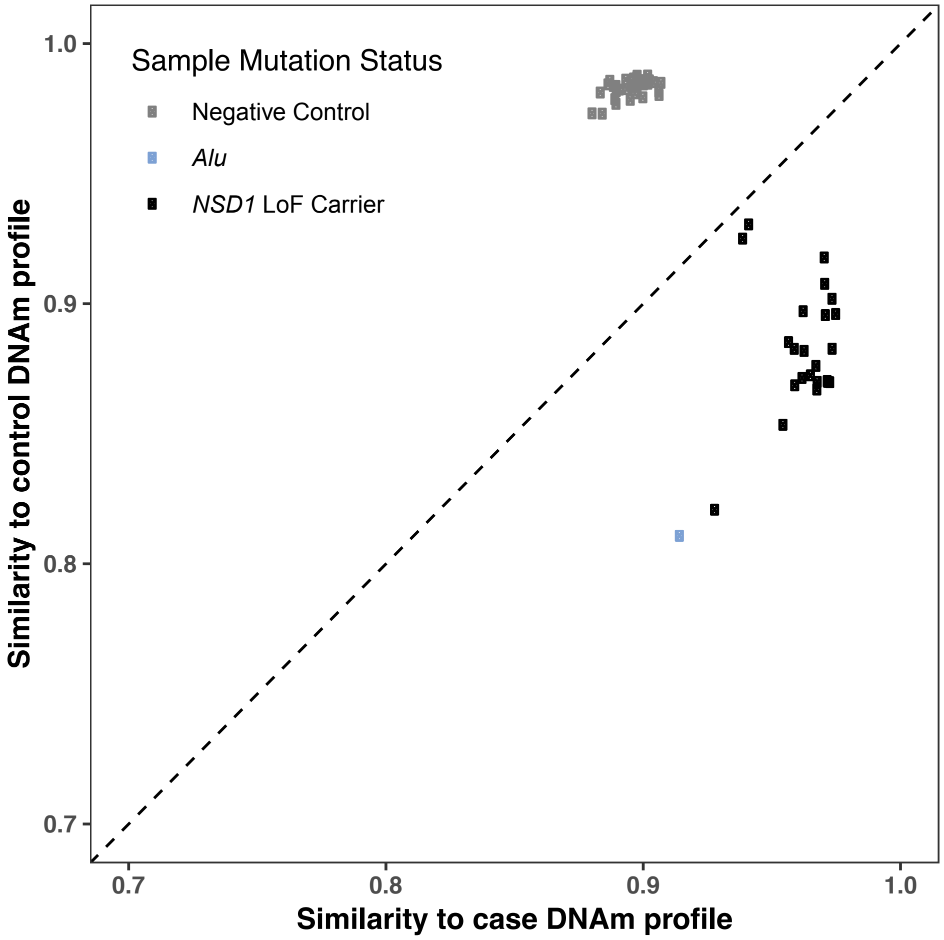

Methylation analysis of individual with NSD1 loss of function *Alu* insertion. Shown are Pearson correlation scores (see methods) to the methylation profile of NSD1 LoF mutation carriers (y-axis) and control individuals (x-axis). The individual with the likely-causative NSD1 *Alu* variant identified in this study is shown in light blue, while case and control individuals from Choufani, et al. [4] are shown as black and grey points, respectively.

Supplemental Figure 11

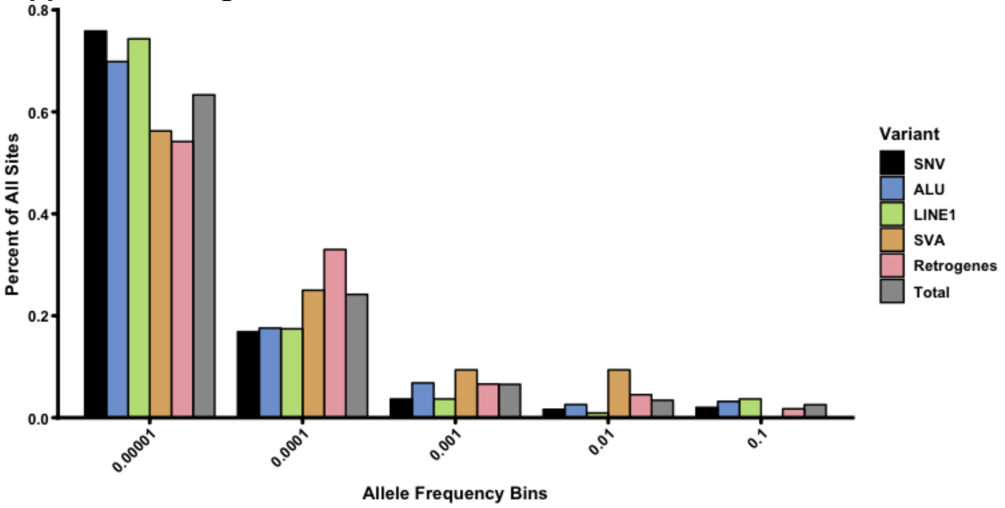

Allele frequency plot in >10X coverage bait regions. Allele frequency plot for all four RT classes, total, and SNVs for comparison. Identical to the plot in main text Fig. 1F, but only for variants within the DDD accessible genome mask (see methods).

Supplemental Figure 12

SLC35F2

PCR primers: 2F and 3R
capillary primer: 2F

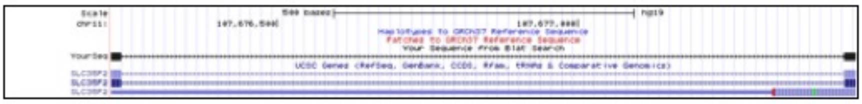

Read Sequence

```
AATCAATACA TCACTCCCTG AATTGTCTTC CCTCCCTccc tgcta
```

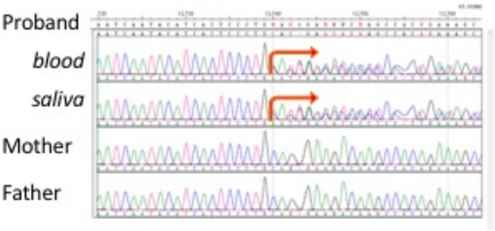

Aligned to Hg19 Sequence

```
acatttgaaa tggcatagag ggaagcccca agaaggacca agatgtcacc  107676224
AATCAATACA TCACTCCctg caggaagaca agccacagaa agcaaaacac  107676274
tgtcaacaga tgaacctcca cagcgtgcct ccaggcggtc atctgactcg  107676324
                         //
ttgatataaa agtgaaaaca taaacaaatt tgctgtgatt gaagcacaaa  107677424
catgtgacac cgtctcacCT GAATTGTCTT CCCTCCCTgc tagtatgtct  107677474
gcaccaacca tggttcctac acccaacaga cagacagcca cggcgatgaa  107677524
```

SERINC5

PCR primers: 2F and 3R
capillary primer: 2F

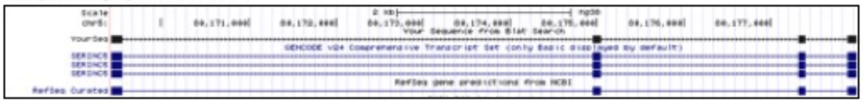

Read Sequence

```
CTGTCTTTCT GTGTATAAAA CACTGCCATC AAAACCAAGC CTCCAGTGGC  50
AATGGAATAC ATGATGAGCG TCACCAGGGC CAGGGAGGCG TACCACAGCT  100
TGTTACTGGC TGTGCCTGCT GTCCAGTTCT TGTTCCACTT ATGTGCAAAC  150
TCCACGAGCA GGAGGAGCTG GATGCCAATG AAGAGGAAGC CTCCGACGGC  200
TCCCACATAG CGCCAGCCGT TCAGAAAGGT GTCCTGATCT GGAATGAAGA  250
AAGCTCCTGA GCACATGGCC CCCAACAGCA GAAGTTTAAA GAACCAAAAG  300
CCATTGTGAA TATGAGCTCT ACAACTTTTG CTGTTGTTGA TTTTCAAGGT  350
CAGTAGACAG AAGATAAAGA AGAAACAAGC CATTCCAAAA CAGACTCTAT  400
ACA
```

Aligned to Hg19 Sequence

```
CTGTCTTTCT GTGTATAAAA CACTGCCATC AAAACCAAGC CTCCAGTGGC  80169473
AATGGAATAC ATGATGAGCG TCACCAGGGC CAGGGAGGCG TACCACAGCT  80169523
TGTTACTGGC TGTGCCTGCT GTCctgtttc tccgggaagt gggtaagagg  80169573
gagaggagag aagacaagtc aacgaagccc accattctgc ggtaacagtc  80169623
                         //
gcaatgcagt tgagtacagc cttggtcaaa actgggcagt ttctgtgagt  80174923
caatgggaag ctttccataa aggcacacac CAGTTCTTGT TCCACTTATG  80174973
TGCAAACTCC ACGAGCAGGA GGAGCTGGAT GCCAATGAAG AGGAAGCCTC  80175023
CGACGGCTCC CACATAGCGC CAGCctgcaa aagaccatga agaacacaat  80175073
                         //
ctatcagagg tactttggac tgcgcttctg agcaatttga caaaatgggt  80177273
aaaaacaaaa taaacagata cccaaaatac cccattagta cCGTTCAGAA  80177323
AGGTGTCCTG ATCTGGAATG AAGAAAGCTC CTGAGCACAT GGCCCCCAAC  80177373
AGCAGAAGTT TAAAGAACCA AAAGctagaa gtgggggaa aaaaaagagg  80177423
aaatgtattt aaatgacatt caagaaggac aaaggacctc gtagaaggta  80177473
                         //
tggactactg tcctgaacat aagaaaggag aaagcaatcc ccaagaagac  80177873
taaaatactt acCCATTGTG AATATGAGCT CTACAACTTT TGCTGTTGTT  80177923
GATTTTCAAG GTCAGTAGAC AGAAGATAAA GAAGAAACAA GCCATTCCAA  80177973
AACAGACTCT ATACAcggca gaatatccca ccagcttctc acaggtgtca  80178023
```

Processed pseudogene (PPG) information sheets. For each *de novo* PPG identified as part of this study, we have collated: primers used (Supplemental Table 3) sequence of the capillary trace (Read Sequence), the resulting trace aligned to the Hg19 human reference genome (Aligned to Hg19 sequence), a graphical representation of the alignment in the UCSC genome browser[8] and, where PCR amplified both PPG and host gene, the intensity data indicating presence of multiple bands in the capillary sequencing.

Supplemental Table 1

Alu					
		WGS			
		0/0	0/1v	1/1	./.
WES	N.P.	234559	65405	16006	221
	N.D.	760	65	47	7
	0/0	2295	123	4	1
	0/1	12	944	61	1
	1/1	0	26	258	0
	./.	51	3	1	0

**L1**					
		WGS			
		0/0	0/1	1/1	./.
WES	N.P.	36944	7232	2236	44
	N.D.	158	9	0	0
	0/0	458	8	0	0
	0/1	1	132	8	0
	1/1	0	0	20	0
	./.	0	0	0	0

**SVA**					
		WGS			
		0/0	0/1	1/1	./.
WES	N.P.	12758	2624	137	25
	N.D.	411	7	1	0
	0/0	55	1	0	0
	0/1	0	1	0	0
	1/1	0	0	0	0
	./.	0	0	0	0

Genotype matricies of matched WGS and WES data. Shown individually for each ME are genotype matrices for sites identified in both WES (left) and WGS (top) data. N.P. (Not possible) are genotypes that were <10x coverage in the matched WES sample and thus were not expected to be identifiable by MELT. N.D. (Not detected) are genotypes that were identified in the WGS data that were >10x coverage in the matched WES sample and should have been identified by MELT. Highlighted in yellow are the matched genotypes used for the genotype accuracy calculation listed in the main text.

Supplemental Table 2

Insertion Coord.	RT Type	HGNC Gene ID	HPO Terms	HPO Phenotypes
**chr3:9495459**	***Alu***	**SETD5**	**HP:0000220|HP:0000431|HP:0000455|HP:0000846|HP:0001161|HP:0001263|HP:0001830|HP:0002205|HP:0002342|HP:0006136|HP:0008915|HP:0100000**	**Adrenal insufficiency|Bilateral postaxial polydactyly|Broad nasal tip|Childhood-onset truncal obesity|Early onset of sexual maturation|Global developmental delay|Hand polydactyly|Intellectual disability|moderate|Postaxial foot polydactyly|Recurrent respiratory infections|Velopharyngeal insufficiency|Wide nasal bridge**
**chr5:176638159**	***Alu***	**NSD1**	**HP:0000238|HP:0000256|HP:0001249|HP:0001250|HP:0001252|HP:0001382|HP:0000729|HP:0010864|HP:0000540|HP:0000733|HP:0001763|HP:0200006|HP:0001263**	**Hydrocephalus|Intellectual disability|Joint hypermobility|Macrocephaly|Muscular hypotonia|Scoliosis|Autistic Behaviour|Severe ID|Hypermetropia|Steryotypy|Pes planus|Slanting of the palpebral fissures|Global developmental delay**
**chr12:46246325**	**L1**	**ARID2**	**HP:0000154|HP:0000176|HP:0000316|HP:0000347|HP:0000475|HP:0000520|HP:0000586|HP:0000960|HP:0001476|HP:0001601|HP:0002020|HP:0002209|HP:0008434|HP:0008872**	**Broad neck|Delayed closure of the anterior fontanelle|Feeding difficulties in infancy|Gastroesophageal reflux|Hypertelorism|Hypoplastic cervical vertebrae|Laryngomalacia|Micrognathia|Proptosis|Sacral dimple|Shallow orbits|Sparse scalp hair|Submucous cleft hard palate|Wide mouth**
**chr5:88100580**	**L1**	**MEF2C**	**HP:0000750|HP:0000915|HP:0010864**	**Delayed speech and language development|Intellectual disability, severe|Pectus excavatum of inferior sternum**

Patient phenotypes and HPO terms. Listed are HPO terms and matched phenotypes as determined by the referring clinician for patients with an identified, clinically actionable MEI. Insertion coord. Is the Hg19 insertion site as listed in main text Table 2.

Supplemental Table 3

Dataset	MEI	Mask	Mask Size	# of haplotypes	# Seg Sites	Theta (Θ)	Effective Populaton Size (N_e)	Genome Mutation Rate ($\mu = \Theta$ / $4*N_e$)	Mutation Rate (μ)
DDD	*Alu*	Exome	74.2E+6bp	34064	653	59.30	10000	1.48E-3	9.99E-12
DDD	L1	Exome	74.2E+6bp	34064	107	9.72	10000	2.43E-4	1.64E-12
DDD	SVA	Exome	74.2E+6bp	34064	30	2.72	10000	6.81E-5	4.59E-13
1KGP	*Alu*	Noncoding	11.1E+8bp	4906	8554	942.56	10000	2.36E-2	1.06E-11
1KGP	L1	Noncoding	9.6E+8bp	4906	2047	225.56	10000	5.64E-3	2.94E-12
1KGP	SVA	Noncoding	9.6E+8bp	4906	329	36.252	10000	9.06E-4	4.72E-13

MEI Mutation rate calculations. Raw data for MEI mutation rate estimates with individuals values used as input to the Watterson estimator[5]. Dataset indicates which dataset was used for the calculation (DDD – this study; 1KGP – Gardner et. al[6]) with Mask indicative of the genome mask used to filter sites (see online methods).

Supplemental Table 4

**Gene**	**Patient Decipher ID**	**Gene Median RPKM**	**Gene RPKM Percentile**
SETD5	280818	10.00	65.3
NSD1	259118	9.26	63.2
ARID2	264759	4.36	38.5
MEF2C	285645	84.37	96.7

Fetal Brain Expression Data. Gene RPKM and percentile values as calculated from fetal brain samples 15 to 37 weeks post-conception from the BrainSpan consortium[7].

Supplementary References

1. Thorvaldsdottir, H., Robinson, J.T. & Mesirov, J.P. Integrative Genomics Viewer (IGV): high-performance genomics data visualization and exploration. *Brief Bioinform* **14**, 178-92 (2013).
2. Zhang, Y., Li, S., Abyzov, A. & Gerstein, M.B. Landscape and variation of novel retroduplications in 26 human populations. *PLoS Comput Biol* **13**, e1005567 (2017).
3. GTEx Consortium. Human genomics. The Genotype-Tissue Expression (GTEx) pilot analysis: multitissue gene regulation in humans. *Science* **348**, 648-60 (2015).
4. Choufani, S. *et al.* NSD1 mutations generate a genome-wide DNA methylation signature. *Nat Commun* **6**, 10207 (2015).
5. Watterson, G.A. On the number of segregating sites in genetical models without recombination. *Theor Popul Biol* **7**, 256-76 (1975).
6. Gardner, E.J. *et al.* The Mobile Element Locator Tool (MELT): population-scale mobile element discovery and biology. *Genome Res* **27**, 1916-1929 (2017).
7. Li, M. *et al.* Integrative functional genomic analysis of human brain development and neuropsychiatric risks. *Science* **362**(2018).
8. Tyner, C. *et al.* The UCSC Genome Browser database: 2017 update. *Nucleic Acids Res* (2016).
