# Supplementary material for: Contribution of retrotransposition to developmental disorders
Source: Nat Commun. 2019 Oct 11;10:4630. doi: 10.1038/s41467-019-12520-y (PMC6789007; doi:10.1038/s41467-019-12520-y)
Supplement: Supplementary file 3 — Description of Additional Supplementary Files [file 41467_2019_12520_MOESM3_ESM.pdf]

Description of Additional Supplementary Files

File Name: Supplementary Data 1
Description: Primers for validation of de novo MEIs

File Name: Supplementary Data 2
Description: Primers and analysis of potential mosaic MEIs
