# Supplementary material for: Contribution of retrotransposition to developmental disorders
Source: Nat Commun. 2019 Oct 11;10:4630. doi: 10.1038/s41467-019-12520-y (PMC6789007; doi:10.1038/s41467-019-12520-y)
Supplement: Supplementary file 6 — Reporting Summary [file 41467_2019_12520_MOESM6_ESM.pdf]

nature research

Corresponding author(s): Matthew Hurles

Last updated by author(s): Jul 31, 2019

Reporting Summary

Nature Research wishes to improve the reproducibility of the work that we publish. This form provides structure for consistency and transparency in reporting. For further information on Nature Research policies, see Authors & Referees and the Editorial Policy Checklist .

Statistics

For all statistical analyses, confirm that the following items are present in the figure legend, table legend, main text, or Methods section.

n/a	Confirmed	
☐	☒	The exact sample size (*n*) for each experimental group/condition, given as a discrete number and unit of measurement
☐	☒	A statement on whether measurements were taken from distinct samples or whether the same sample was measured repeatedly
☐	☒	The statistical test(s) used AND whether they are one- or two-sided<br>*Only common tests should be described solely by name; describe more complex techniques in the Methods section.*
☐	☒	A description of all covariates tested
☐	☒	A description of any assumptions or corrections, such as tests of normality and adjustment for multiple comparisons
☐	☒	A full description of the statistical parameters including central tendency (e.g. means) or other basic estimates (e.g. regression coefficient) AND variation (e.g. standard deviation) or associated estimates of uncertainty (e.g. confidence intervals)
☐	☒	For null hypothesis testing, the test statistic (e.g. *F*, *t*, *r*) with confidence intervals, effect sizes, degrees of freedom and *P* value noted<br>*Give P values as exact values whenever suitable.*
☒	☐	For Bayesian analysis, information on the choice of priors and Markov chain Monte Carlo settings
☒	☐	For hierarchical and complex designs, identification of the appropriate level for tests and full reporting of outcomes
☒	☐	Estimates of effect sizes (e.g. Cohen's *d*, Pearson's *r*), indicating how they were calculated

Our web collection on statistics for biologists contains articles on many of the points above.

Software and code

Policy information about availability of computer code

Data collection	For MEI Calling, publicly available MELTv2.1.5. For Retrogene calling, custom code available on github as stated in manuscript methods. SNVs were called with the Genome Analysis Toolkit v3.5.
Data analysis	All R code and intermediate files used to generate figures will be made publicly available on github pending publication.

For manuscripts utilizing custom algorithms or software that are central to the research but not yet described in published literature, software must be made available to editors/reviewers. We strongly encourage code deposition in a community repository (e.g. GitHub). See the Nature Research guidelines for submitting code & software for further information.

Data

Policy information about availability of data

All manuscripts must include a data availability statement. This statement should provide the following information, where applicable:

- Accession codes, unique identifiers, or web links for publicly available datasets
- A list of figures that have associated raw data
- A description of any restrictions on data availability

All DDD sequencing data is available under EGA study ID EGAS00001000775. VCF files of RT variant calls are available as part of this accession EGAD00001004390 and EGAD00001004586

Field-specific reporting

Please select the one below that is the best fit for your research. If you are not sure, read the appropriate sections before making your selection.

☒

Life sciences study design

All studies must disclose on these points even when the disclosure is negative.

Sample size	Samples utilized in this study included all patients recruited for the DDD study that were sequenced as part of a trio (i.e. patient along with mother and father) representing an n = 28,132 non-redundant individuals.
Data exclusions	For the purposes of this study, individuals that were included on the DDD sample blacklist due to sequencing QC fails or failed to meet MELT QC requirements were excluded from downstream analysis.
Replication	All variant calls reported in this study were determined with either previously validated tools or with tools validated as part of this study using a comparison to previous studies. All de novo variants reported in the manuscript were confirmed independently via PCR. Mutation rates were validated via comparison to previously published variants from the 1000 Genomes Project as well as previous work by other independent groups.
Randomization	Patients were selected based on clinical assessment for the DDD study and unaffected parents were determined based on HPO terms recorded at the time of patient consent. We did not randomize into groups as part of this study.
Blinding	Variants were ascertained independent of any clinical phenotypes and were reported blind to clinicians for confirmation.

Reporting for specific materials, systems and methods

We require information from authors about some types of materials, experimental systems and methods used in many studies. Here, indicate whether each material, system or method listed is relevant to your study. If you are not sure if a list item applies to your research, read the appropriate section before selecting a response.

Materials & experimental systems

n/a	Involved in the study	
☒	☐	Antibodies
☒	☐	Eukaryotic cell lines
☒	☐	Palaeontology
☒	☐	Animals and other organisms
☐	☒	Human research participants
☒	☐	Clinical data

Methods

n/a	Involved in the study	
☒	☐	ChIP-seq
☒	☐	Flow cytometry
☒	☐	MRI-based neuroimaging

Human research participants

Policy information about studies involving human research participants

Population characteristics	Primary selection was done on the presentation of a patient with a developmental disorder phenotype. Alongside patients, in most cases, parents were recruited into the study as well. As such, population characteristics are quite broad.
Recruitment	DDD was recruited from developmental disorder patients throughout the UK and Republic of Ireland. Patients were ascertained on the basis of an as-then indeterminable developmental disorder phenotype. As such, our findings tend to reflect identification of disorders less recognizable on initial clinical presentation.
Ethics oversight	The study has UK Research Ethics Committee approval (10/H0305/83, granted by the Cambridge South REC, and GEN/284/12 granted by the Republic of Ireland REC)

Note that full information on the approval of the study protocol must also be provided in the manuscript.
